# Mitochondrial replacement techniques and Mexico's rule of law: on the legality of the first maternal spindle transfer case

**DOI:** 10.1093/jlb/lsx035

**Published:** 2017-10-13

**Authors:** César Palacios-González, María de Jesús Medina-Arellano

*Journal of Law and the Biosciences* **4**:1 2017. doi: 10.1093/jlb/lsw065

Following the publication of this article, new information became available, regarding where the first case of mitochondrial replacement therapy discussed in this article was carried out. In a publication, Dr. John Zhang and his team revealed that ‘the ovarian stimulation cycles and oocyte manipulations were carried out at a private fertility clinic in New York, the vitrified embryo was then shipped to Mexico to be warmed and transferred to the patient at an affiliate clinic in Guadalajara’.

When we examined the legality of this case, we did so according to the information available to us at the time. In a response published on October 13^th^, 2017 we provide further details and discuss further implications of this.

John Zhang et al., Live birth derived from oocyte spindle transfer to prevent mitochondrial disease, 34 REPROD.BIOMED.ONLINE 361–368 (2017).

